# Nutritional, Rheological, and Sensory Assessment of Functional Toast Bread Fortified With Rye Flour and Basil Seed Gum Powder

**DOI:** 10.1155/ijfo/8841214

**Published:** 2025-11-19

**Authors:** Milad Daneshniya, Mohammad Hossein Maleki, Farzaneh Abdolmaleki, Zahra Latifi

**Affiliations:** ^1^ Department of Food Science and Technology, Qa.C., Islamic Azad University, Qazvin, Iran, azad.ac.ir; ^2^ Department of Food Science and Technology, SR.C., Islamic Azad University, Tehran, Iran, azad.ac.ir; ^3^ Department of Food Science and Technology, Nour C., Islamic Azad University, Mazandaran, Iran, azad.ac.ir

**Keywords:** basil seed gum, functional toast, rheological property, rye flour, sensory property

## Abstract

The growing demand for functional foods has led to the exploration of alternative ingredients to enhance the nutritional and sensory qualities of bread. This study investigates rye flour (RF) and basil seed gum powder (BSGP) in functional toast bread, evaluating their impact on chemical composition, dough rheology, texture, and sensory properties. BSGP, extracted from basil seeds, was combined with RF at varying concentrations (0%, 15%, and 25% RF and 0%, 0.5%, and 1% BSGP) to produce six different formulations. Chemical analysis revealed that increasing RF and BSGP concentrations significantly enhanced moisture, ash, fat, and fiber content, with the highest concentrations (T6: 25% RF, 1% BSGP) showing the most substantial improvements. Rheological analysis indicated that BSGP reduced pasting temperature and peak viscosity, while higher RF concentrations increased adhesiveness and brittleness over time. Textural evaluations demonstrated that 1% BSGP optimally maintained springiness, while higher RF levels negatively affected texture, resulting in increased brittleness. Sensory analysis identified the combination of 0.5% BSGP and 15% RF (T3) as the most acceptable, balancing taste, texture, and color. Storage studies revealed that Day 1 samples exhibited higher moisture and springiness, whereas Day 5 samples showed increased hardness, brittleness, and adhesiveness, particularly in formulations with higher BSGP levels. Principal component analysis (PCA) captured these temporal trends, emphasizing the relationship between moisture, elasticity, and firmness. In conclusion, RF and BSGP enhance bread′s nutritional and sensory properties but require precise formulation to optimize fresh and stored product quality.

## 1. Introduction

In recent years, there has been growing interest in developing functional foods that offer health benefits beyond basic nutrition [1–3]. Among these, functional bread products have attracted considerable attention for their ability to incorporate health‐promoting ingredients without compromising sensory qualities [4]. Toast bread, in particular, stands out as an ideal candidate for fortification due to its widespread consumption and broad appeal across all age groups [5, 6]. Its soft texture, mild flavor, and versatility—commonly enjoyed at breakfast or in sandwiches—make it highly acceptable, even among children and older adults [7]. Furthermore, the uniform structure of toast bread supports the seamless integration of functional ingredients such as fiber‐ and protein‐rich by‐products, while maintaining desirable texture and taste [8]. Its relatively long shelf life and suitability for large‐scale production also enhance its practicality for public health interventions [7]. By fortifying toast bread, manufacturers can not only improve its nutritional value but also meet growing consumer demand for healthier, convenient, and affordable food options.

Research on alternative flours for bread production has explored various aspects of ingredient composition and processing techniques. Partial substitution of refined wheat flour with wheat bran or whole grain wheat flour increases water absorption and crumb firmness but decreases bread volume [9]. For rice flour bread, low damaged starch content and optimal amylose levels (16%–20%) are crucial for quality [10]. Whole wheat flour bread exhibits superior antioxidant properties compared to refined versions, although baking affects these properties differently across various antioxidant measures [11]. Techniques such as soaking nonflour milling fractions, adding salts and surfactants, and incorporating enzyme‐active soy flour have been investigated to improve whole wheat bread volume [12]. These studies highlight the importance of understanding flour characteristics and processing methods to optimize bread quality and nutritional value in wheat‐ and rice‐based products. Among the various functional flours studied, rye flour (RF) has also emerged as a promising component due to its high fiber content, bioactive compounds, and potential to improve digestive health and glycemic control [13]. Rye is also rich in phenolic acids, contributing to its antioxidant properties, making it a valuable ingredient for functional bakery products [14].

In addition to RF, basil seed gum powder (BSGP), a hydrocolloid derived from *Ocimum basilicum* seeds, is increasingly being explored for its functional and technological properties in food systems. The BSGP has shown promising effects in improving bread quality. Studies have demonstrated that BSGP addition can significantly enhance bread volume, specific volume, and width/height ratio while reducing weight loss [15, 16]. BSGP improves bread microstructure and porosity, producing smoother surfaces with fewer cavities [15]. In gluten‐free bread, BSGP acts as a gluten substitute, mimicking its viscoelastic properties [17]. Adding BSGP increases dough viscosity and exhibits pseudoplastic and thixotropic behavior [16]. BSGP also affects starch gelatinization and retrogradation, leading to softer bread products with extended shelf life [17]. Sensory evaluations have shown favorable results for bread containing BSGP, with optimal concentrations ranging from 1% to 2% [15, 16]. Overall, BSGP proves to be an effective hydrocolloid for improving the nutritional, physicochemical, and sensory profiles of bread.

The combination of RF and BSGP holds promise for enhancing both the nutritional and sensory qualities of toast bread [18, 19]. While RF contributes valuable nutrients and bioactive compounds, its inclusion may lead to undesirable changes in texture and sensory attributes, reducing consumer acceptance [20, 21]. In contrast, BSGP, a natural hydrocolloid, has shown potential to improve dough structure, bread volume, and softness [22]. However, the combined use of RF and BSGP in toast formulations has not been systematically studied. Previous research has examined RF and BSGP separately, but there is limited understanding of their interactive effects on bread′s rheological, nutritional, and sensory properties. This represents a key gap in functional bakery product development.

This study sought to bridge the identified research gap by systematically evaluating the effects of varying incorporation levels of RF and BSGP on the physicochemical, rheological, nutritional, and sensory attributes of functional toast bread formulations. Specifically, the primary objective was to elucidate the synergistic interactions between RF and BSGP, thereby identifying optimal blend ratios that maximize nutritional fortification—such as enhanced fiber, protein, and bioactive compound content—while mitigating adverse impacts on dough rheology, bread volume, crumb texture, and overall consumer acceptability. Through this approach, the investigation contributes to the development of innovative, health‐promoting bakery products that align with contemporary demands for nutrient‐dense, sensory‐appealing alternatives to conventional wheat‐based breads.

## 2. Materials and Methods

### 2.1. Preparation of BSGP and RF

The basil seeds were bought in commercially available packages for culinary purposes from the reputable local market (Qazvin, Iran). A 500‐g pack of seeds was cleaned using a 30‐mesh sieve (Merck, Germany) to remove impurities. The seeds were then soaked in distilled water (pH 7) at 25°C for 20 min with a water‐to‐seed ratio of 20:1 (*v*/*w*). The gum was extracted using a mill (Panasonic, Japan), separated from the seeds, and dried in an oven at 105°C for 4 h. The dried gum was ground and passed through a 50‐mesh sieve (Merck, Germany). The yielded BSGP was stored in moisture‐ and oxygen‐resistant insulated bags at room temperature [23]. In addition, the RF with 9.5% moisture, 10.4% protein, 1.76% fat, and 1.6% ash content was purchased from the Zagros Jahan Co. (Chaharmahal Bakhtiari, Iran).

### 2.2. Bread Production

To produce toasted bread, the required raw materials were first prepared and weighed, including wheat flour, salt, sugar, water, *Saccharomyces cerevisiae* yeast, and oil. Additionally, according to Table 1, RF was incorporated at levels 0%, 15%, and 25%, along with BSGP, at levels of 0%, 0.5%, and 1.0% of the wheat flour weight.

**Table 1:** Composition of the ingredients utilized in the preparation of the bread samples.

**Treatment**	**Wheat flour (g)**	**Salt (g)**	**Sugar (g)**	**Water (g)**	**Yeast (g)**	**Oil (g)**	**BSGP (g)**	**RF (g)**
T	1000	15	30	600	20	30	0	0
T1	1000	15	30	600	20	30	0	150
T2	1000	15	30	600	20	30	0	250
T3	1000	15	30	600	20	30	5	150
T4	1000	15	30	600	20	30	5	250
T5	1000	15	30	600	20	30	10	150
T6	1000	15	30	600	20	30	10	250

This allowed for varying formulations to assess the effects of these ingredients on the final product. Regarding the mentioned ratio of each BSGP and RF, it should be noted that these ratios are the outcome of the pretest, primarily focused on the sensory and textural properties of the bread; so the ratios in individual form—for Treatments 1 and 2—and in combination—for Treatments 3 to 6—did not imply significant negative textural and sensory impact. The dough was prepared in a dough tank, where the ingredients were mixed for about 20 min until a moldable dough mass was achieved. Once combined, the dough samples were subjected to a 10‐min relaxation period. After this initial rest, the dough was divided into pieces weighing approximately 450 g, rounded, and underwent a 10‐min bench rest. The dough was baked in an oven at three different temperatures—170°C, 180°C, and 200°C (the maximum threshold)—each for 45 min, to assess the effect of baking temperature on product quality. Based on the results of preliminary textural analyses, 170°C was identified as the optimal baking temperature and was therefore selected to prepare all subsequent experimental samples. After preparing the bread treatments, the evaluations were conducted on each treatment with three replicates, followed by expressing the final result as an average plus standard deviation.

### 2.3. Physicochemical Properties of the Bread

The physicochemical properties of bread are critical determinants of consumer acceptability. In this study, all physicochemical parameters were evaluated on the day of production, with the exception of moisture content, springiness, adhesiveness, hardness, and brittleness, which were additionally assessed after 5 days of storage to examine the impact of storage duration on bread quality. During storage, the bread samples were kept in sealed polyethylene bags at ambient temperature (approximately 25^°^C ± 2^°^C) under dark, dry conditions to minimize moisture loss and light‐induced changes.

#### 2.3.1. Ash Measurement

The electric furnace method was utilized to measure the ash content. Initially, a porcelain crucible was preheated in an electric furnace (Shimiazma, Iran) at 500°C for 30 min, followed by cooling in a desiccator before being weighed. Subsequently, 2 g of the sample was placed in the crucible, preburned over an open flame, and then transferred to the electric furnace. The sample was incinerated at 576°C for 6 h, according to AACC, Method 08‐01 [24]. After the process, the ash content was determined by weighing the remaining residue according to the equation below:

(1) Ash %=Wb−WcWs×100,

where *W*_*b*_ represents the weight of the crucible with ash, *W*_*c*_ represents the weight of the empty crucible, and *W*_*s*_ represents the sample weight.

#### 2.3.2. Moisture Measurement

A particular metal container was first placed in an oven at 130^°^C ± 2^°^C for 30 min to measure moisture content. After this, the container was cooled in a desiccator to room temperature and weighed. A 2‐g sample was placed into the container and transferred back to the oven. After 1 h, the container was removed from the oven, cooled in the desiccator, and weighed again. This process was repeated until a constant weight was achieved. The moisture percentage was then calculated using the formula specified by AACC Method 44‐19 [25]:

(2) Moisture %=Wpre−WpostWs×100,

where *W*_*p* *r* *e*_ and *W*_*p* *o* *s* *t*_ represent the weights of the sample before and after drying, respectively, and *W*_*s*_ is the sample weight.

#### 2.3.3. Protein Content Evaluation

The micro‐Kjeldahl method, highlighted by Maleki et al. [1], with modifications, was employed to measure the protein content, which involved two primary stages: digestion and distillation. During digestion, 0.5 g of a thoroughly homogenized sample was combined with half a catalyst tablet containing 150 mg of copper sulfate and 150 mg of selenium powder. Additionally, 15 mL of concentrated sulfuric acid was added to the flask, and the mixture was gently heated until the solution′s color lightened. After digestion, the solution was allowed to cool and was diluted to 50 mL in a volumetric flask. A control flask (without the sample) underwent the same procedure for comparison.

In the distillation stage, 15 mL of 4% boric acid, along with two drops of methyl red indicator, was placed in an Erlenmeyer flask and connected to the condenser of the distillation apparatus. Next, 10 mL of 50% alcohol was added to the distillation apparatus. After distillation for 15 min, the distillate was titrated using 0.02 M hydrochloric acid. The protein percentage was then calculated using the following equation, where several factors were included: the volume of acid used for the sample (*V*), the volume of acid for the control (*V*_1_), the normality of hydrochloric acid (*N*), the nitrogen‐to‐protein conversion factor (*F*), dilution/aliquot factor (*D*), and the sample weight (*W*):

(3) %Protein=V−V1×N×14.007100×F×D×W×100.

#### 2.3.4. Fat Content Measurement

The Soxhlet extraction method was employed to determine the fat content. A 2‐g sample, uniformly dried and wrapped in filter paper, was placed inside a paper thimble, which was then positioned in a specialized Soxhlet apparatus (Shimiazma, Iran). The thimble was connected to a balloon containing 150 mL of hexane solvent, and the system was gently heated for 6–8 h to facilitate fat extraction. The temperature was maintained at 60°C to ensure proper solvent evaporation. Once the extraction process was complete, the balloon was cooled in a desiccator. The fat percentage was then calculated based on the weight of the extracted fat using the formula according to AACC Method 30‐25 [26] with modifications:

(4) Fat %=Wff−WefWs×100,

where *W*_*f* *f*_ is the weight of the flask and fat, *W*_*e* *f*_ is the weight of the empty flask, and *W*_*s*_ is the sample weight.

#### 2.3.5. Dough Viscosity Evaluation

The viscosity was determined using a Rapid Visco Analyzer (RVA). This device was used to assess the kneading characteristics of flour samples mixed with BSGP. First, the samples were finely ground and passed through an 80‐mesh sieve. The dough of each treatment was poured into an aluminum container and stirred with a plastic paddle for 20–30 s before being placed in the RVA (PerkinElmer, United States). The device settings included an initial speed of 960 revolutions per minute for the first 10 s, followed by a constant speed of 16 revolutions per minute. The initial temperature was set at 50°C, the temperature was held steady at 50°C for the first minute, increased linearly to 95°C over 4 min and 48 s, maintained at 95°C for 7 min and 18 s, reduced linearly back to 50°C by 11 min and 6 s, and finally held constant at 50°C until a total of 12 min and 30 s had elapsed.

#### 2.3.6. Bread Physical Characteristic Measurement

The rapeseed displacement method was employed according to AACC Method 10‐05.01 to measure the volume of different bread treatments [27]. Initially, the empty container was weighed, and its volume was determined by filling it with water. The container was then completely filled with rapeseed seeds. The seeds′ surface was leveled using a long ruler drawn across the top of the container. After weighing the filled container, the density of the rapeseed seeds was calculated using the following equation:

(5) ρ=Wcs−WecV.

In this equation, *W*_*c* *s*_ represents the weight of the container with rapeseed, and *W*_*e* *c*_ represents the weight of the empty container, both measured in grams. The weight of the rapeseed was calculated by subtracting *W*_*e* *c*_ from *W*_*c* *s*_, and the volume of the container (*V*) was measured in cm^3^. The density of the rapeseed, *ρ*, was then expressed in cm^3^.

Subsequently, the preweighed bread samples were placed individually in the same container, which was then filled with rapeseed seeds, ensuring even distribution. After weighing the container with the bread and rapeseed, the volume and density of the bread were determined using the following equations according to AACC Method 10‐90 [28]:

(6) VA=Wcbr−Wcbρ,

(7) Vb=Vt−Vr,

(8) ρb=WbVb.

In these equations, *W*_*c* *b* *r*_ represents the weight of the container with bread and rapeseed, and *W*_*c* *b*_ represents the weight of the container with only the bread, both in grams. The previously calculated rapeseed density, *ρ*, was used to determine the rapeseed volume displaced by the bread *V*_*r*_. The bread volume *V*_*b*_ was calculated by subtracting *V*_*r*_ from the total container volume (*V*_*t*_). Finally, the bread density (*ρ*_*b*_) was determined by dividing the bread weight (*W*_*b*_) by the bread volume (*V*_*b*_).

The specific volume of the bread, in cm^2^/g, was calculated as the inverse of the density following the guidelines of AACC Method 72‐10 [29]:

(9) SV=1ρ.

#### 2.3.7. Bread Texture Evaluation

The texture of the bread crust and core was evaluated using a rheological method, according to Ren et al. [30], with modifications, a common approach for measuring bread staleness. A texture analyzer (ENCO, Italy) was used to conduct the test, which provided data on the force required to achieve a specific level of compression or the extent of compression caused by a standard force. The test involved placing cut pieces of bread on a holding plate, where a probe moved back and forth over the samples. Based on the time elapsed, the level of indentation, and the power consumed, a diagram was generated. From this diagram, several parameters were extracted, including hardness, adhesion, consistency, chewability, and other key textural properties.

The textural properties of the bread were analyzed on different days after baking, specifically on the first and fifth days, with all samples stored at 25°C. The aim was to assess changes in the bread′s stiffness, cohesion, adhesion, and springiness over time. A cylindrical probe with a 90‐mm diameter was used in the test, moving downward at a speed of 5 mm/s. Upon contacting the bread sample, the probe applied pressure at a speed of 1 mm/s, compressing the sample by 25% of its diameter before moving upward. After a 10‐s interval, the probe applied pressure again at the same speed.

#### 2.3.8. Sensory Evaluation

Sensory characteristics, including color, taste, and texture, were evaluated using the hedonic scale method, according to Latifi et al. [31], with modifications, by 10 trained evaluators. Each evaluator completed a sensory evaluation questionnaire to assess the quality of the bread samples. The hedonic scale ranged from 1 to 9, with 1 representing the lowest score (indicating strong dislike) and 9 representing the highest score (indicating strong preference). In the sensory evaluation, various bread samples were tested based on their formulation, including different levels of BSGP (0%, 0.5%, 1%) and RF (15%, 25%).

#### 2.3.9. Statistical Analysis

The experimental results are expressed as the mean ± SD from three independent replicates of each experiment. Statistical analysis was performed using one‐way analysis of variance (ANOVA), followed by Tukey′s honestly significant difference (HSD) post hoc test to evaluate pairwise comparisons, with a significance threshold set at 0.05 (*p* < 0.05) considered statistically significant. The analysis was conducted using SPSS Statistics (Version 27.0), and graphical representations of the data were generated using Excel software (Version 2020). For the parameters which were evaluated in two intervals—production day and the fifth day of storage—the principal component analysis (PCA) model was used for data projection; PCA was performed using XLSTAT (ver. 2022), with standardization carried out using the *n* − 1 approach to ensure the equal contribution of all variables regardless of their original units. The PCA model was constructed based on the Pearson correlation matrix, with a maximum of five factors considered to explore the dimensional structure of the dataset. Significant components were identified based on eigenvalues greater than 1 (Kaiser′s criterion), and variables with loadings exceeding ±0.5 were considered to contribute meaningfully to the components. A significance level of *α* = 0.05 was used for interpreting the results, ensuring statistical validity.

## 3. Results and Discussion

### 3.1. Parameters Evaluation Conducted on the Day of Production

#### 3.1.1. Ash Content

**Figure 1:**
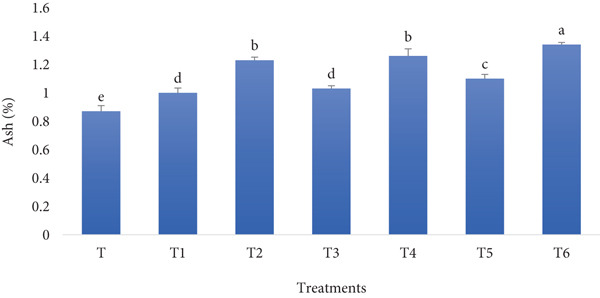
Ash content of functional bread samples prepared with rye flour and basil seed gum powder. Results are indicated as mean ± SD (*n* = 3). Different letters show a significant difference (*p* ≤ 0.05) among the treatments, as assessed by one‐way ANOVA and Tukey′s honestly significant difference tests (T: control, T1: 0% BSGP–15% RF, T2: 0.0% BSGP–25% RF, T3: 0.5% BSGP–15% RF, T4: 0.5% BSGP–25% RF, T5: 1% BSGP–15% RF, T6: 1% BSGP–25% RF).

Ash content indicates the mineral material in the bread, originating primarily from the flour and any added ingredients. Figure 1 reveals the ash content of the different treatments on production day. An increasing trend of the ash content can be seen for the treatment, following a growing percentage of the RF and BSGP in the bread flour.

In the control sample (T), which contained no RF or BSGP, the ash content was the lowest, measuring approximately 0.85%. Adding 0% BSGP and 15% RF (T1) slightly increased the ash content to around 1.03%, but this increase was not statistically significant from T3 (0.5% BSGP–15% RF), which also measured 1.03%. The highest ash content was observed in the T6 treatment (1% BSGP–25% RF), which reached approximately 1.35% and was significantly higher than all other treatments. T2 (0% BSGP–25% RF) and T4 (0.5% BSGP–25% RF) showed relatively higher ash contents of approximately 1.22%, and these values were significantly different from all the treatments. T5 (1% BSGP–15% RF) exhibited an ash content of about 1.15%, substantially lower than T6 but higher than T and T1.

These results indicate that including RF and BSGP in the formulation of functional toast significantly influences the ash content, with higher percentages of both components leading to increased ash levels. This trend suggests that RF, in particular, contributes to the mineral content of the bread, and the synergistic effect with BSGP enhances this outcome. Therefore, the highest ash content was achieved when both BSGP and RF were included at their highest concentrations (T6 1% BSGP–25% RF). In alignment with the findings of the current study, previous investigations have also reported that fortifying bread with various ingredients can significantly enhance its nutritional profile and health benefits. Studies have shown that incorporating buckwheat flour [32]; leafy vegetables like moringa, coriander, and amaranth [33]; and defatted soy flour into wheat bread can increase ash content, protein, fiber, and mineral levels [34]. These fortifications also improved antioxidant properties and reduced glycemic index in some cases [33]. However, the level of fortification must be carefully balanced to maintain acceptable sensory attributes and rheological properties [34]. The mineral content of bread can vary considerably depending on the type of flour used and fortification practices. For instance, rye bread generally has higher mineral content, while French bread may have higher iron levels due to flour fortification [35]. These studies demonstrate the potential of bread fortification to enhance nutritional value while maintaining consumer acceptability.

#### 3.1.2. Protein Content

**Figure 2:**
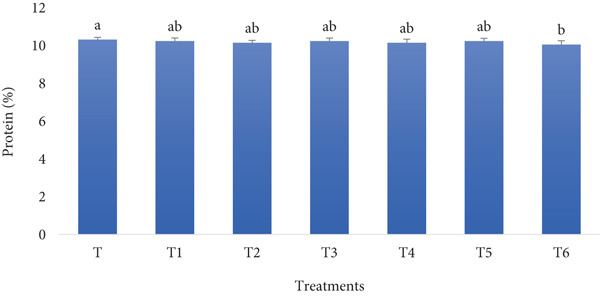
Protein content of functional bread samples prepared with rye flour and basil seed gum powder. Results are indicated as mean ± SD (*n* = 3). Different characters show a significant difference (*p* ≤ 0.05) among the samples, as assessed by one‐way ANOVA and Tukey′s honestly significant difference tests (T: control, T1: 0% BSGP–15% RF, T2: 0.0% BSGP–25% RF, T3: 0.5% BSGP–15% RF, T4: 0.5% BSGP–25% RF, T5: 1% BSGP–15% RF, T6: 1% BSGP–25% RF).

Bread made from fortified flour with RF and BSGP may have slightly lower protein content due to the dilution effect of these ingredients, but it gains enhanced fiber, moisture retention, and overall nutritional value. This formulation prioritizes functional benefits and health improvements, such as better texture and potential prebiotic effects, over maximizing protein levels. As shown by Figure 2, the protein measurement results agree with the prediction, as a slight reduction in the protein content follows the fortification growth.

The control sample (T), which contained no RF or BSGP, exhibited the highest protein content at approximately 10.4%. This was statistically similar to treatments T1 (0% BSGP–15% RF), T2 (0% BSGP–25% RF), T3 (0.5% BSGP–15% RF), T4 (0.5% BSGP–25% RF), and T5 (1% BSGP–15% RF), all of which showed protein contents around 10.2%–10.4%, with no statistically significant differences among these treatments. In contrast, treatment T6 (1% BSGP–25% RF) exhibited a slightly lower protein content of approximately 10.1%, which was statistically different (*p* ≤ 0.05) from the control (T), indicating its significant reduction in protein compared to T.

Overall, including RF and BSGP at the given concentrations did not result in significant variations in protein content among the samples, with most values hovering around 10.2%–10.4%. However, the highest levels of BSGP (1%) and RF (25%) in T6 caused a small but statistically significant reduction in protein content, suggesting a minor dilution effect of BSGP on protein concentration when used at high levels. Despite this, the variations observed among most treatments were relatively minor, indicating that the protein content of these functional toast samples is maintained relatively consistently regardless of the formulation adjustments. As highlighted by previous studies, fortifying bread with various protein sources can significantly enhance its nutritional value. Studies have shown that incorporating tilapia fish protein flour [36], bean flour [37], soy flour, and whey protein concentrate [38] into bread formulations can substantially increase protein content, which is in contrast to the outcome of the present study. The tilapia fish protein flour increased protein content from 9.08% to 18.01% [36], while spirulina powder fortification raised it to 15.43% [39]. These fortifications boost protein levels and improve other nutritional aspects, such as calcium content, dietary fiber, and antioxidant activity [37, 39]. Importantly, fortified breads maintained acceptable organoleptic properties up to certain fortification levels [36, 39]. These studies demonstrate that fortified bread can be a viable strategy to enhance nutritional quality without significantly altering consumer acceptability, which, by considering the ash and protein content of the treatment for the current study, only partially aligns with our findings.

#### 3.1.3. Fat Content

**Figure 3:**
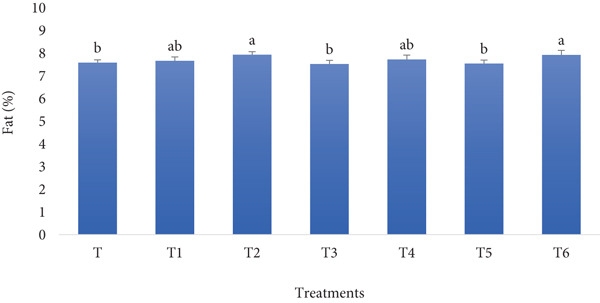
Fat content of functional bread samples prepared with rye flour and basil seed gum powder. Results are indicated as mean ± SD (*n* = 3). Different characters show a significant difference (*p* ≤ 0.05) among the samples, as assessed by one‐way ANOVA and Tukey′s honestly significant difference tests (T: control, T1: 0% BSGP–15% RF, T2: 0.0% BSGP–25% RF, T3: 0.5% BSGP–15% RF, T4: 0.5% BSGP–25% RF, T5: 1% BSGP–15% RF, T6: 1% BSGP–25% RF).

The fortification of the flour of the bread can affect the fat content of the bread, depending on the fat content of the ingredients contributing to fortification. The fat content of bread fortified with RF and BSGP is likely to remain relatively low, as neither ingredient is a significant source of fat. The BSGP contributes primarily fiber, and RF contains minimal fat, so any change in fat content would depend on other ingredients in the formulation rather than these additions. As the fat content evaluation results in Figure 3 support the expectation, the control sample does not differ substantially from fortified treatments.

The control sample (T), which contains no RF or BSGP, exhibited a fat content of approximately 7.0%. This was statistically different from treatments T2 (0% BSGP–25% RF) and T6 (1% BSGP–25% RF), which had the highest fat content at around 7.9%; treatments T1 (0% BSGP–15% RF) and T4 (0.5% BSGP–25% RF) showed intermediate fat contents of about 7.6%, with no significant differences from each other but showing slight variation from different treatments. Meanwhile, T3 (0.5% BSGP–15% RF) and T5 (1% BSGP–15% RF) exhibited fat contents closer to 7.1%, statistically similar to the control but significantly lower than T2 and T6.

Overall, the results indicate that the fat content in these functional toast samples is influenced by both RF and BSGP levels. Treatments with higher RF content (25%) tend to have higher fat percentages, and adding BSGP at 1% in combination with RF at 25% (T6) produced the highest fat content among the samples. These findings suggest that increasing the RF content, particularly in combination with higher BSGP concentrations, has a notable effect on the fat composition of the toast samples. According to previous studies, fortifying bread with various ingredients can be associated with alterations in fat content. Adding winged bean flour increases the protein, fat, calcium, phosphorus, and iron content in bread [40]. Sesame seed fortification improved protein, fiber, and fat content, as well as sensory properties and shelf life [41]. Similarly, buckwheat flour substitution increased ash, protein, fat, fiber, and mineral content, with 30% substitution optimal for nutritional and sensory qualities [32]. These studies demonstrate that fortified bread can effectively improve nutrient intake and address specific dietary deficiencies in populations while maintaining acceptable sensory characteristics. Nevertheless, the current study result revealed not a very outstanding difference between the fat content of the control sample and the treatment with the highest concentration of RF and BSGP.

#### 3.1.4. Fiber Content

**Figure 4:**
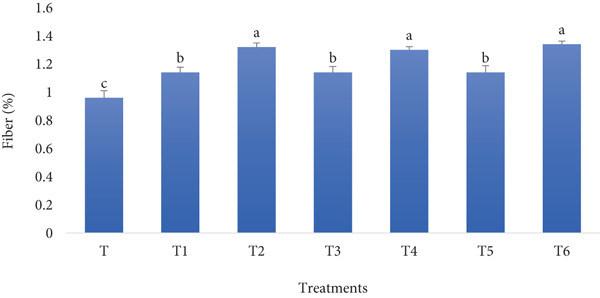
Fiber content of functional bread samples prepared with rye flour and basil seed gum powder. Results are indicated as mean ± SD (*n* = 3). Different characters show a significant difference (*p* ≤ 0.05) among the samples, as assessed by one‐way ANOVA and Tukey′s honestly significant difference tests (T: control, T1: 0% BSGP–15% RF, T2: 0.0% BSGP–25% RF, T3: 0.5% BSGP–15% RF, T4: 0.5% BSGP–25% RF, T5: 1% BSGP–15% RF, T6: 1% BSGP–25% RF).

RF and BSGP are rich sources of fiber. The fiber content of bread fortified with RF and BSGP will likely increase significantly. The RF is a good source of dietary fiber, and BSGP is exceptionally high in soluble fiber, contributing to enhanced gut health and improved bread texture and shelf life. According to Figure 4, the assumption is close to reality.

The control sample (T), which contains no RF or BSGP, exhibited a fiber content of approximately 0.9%. This was statistically different from treatments T2 (0% BSGP–25% RF) and T6 (1% BSGP–25% RF), which had the highest fiber content at around 1.4%. Treatments T1 (0% BSGP–15% RF) and T4 (0.5% BSGP–25% RF) showed intermediate fiber contents of about 1.1% and 1.3%, respectively, with no significant differences from each other but showed slight variation from other treatments. Meanwhile, T3 (0.5% BSGP–15% RF) and T5 (1% BSGP–15% RF) exhibited fiber contents closer to 1.1%, statistically similar to the control but significantly lower than T2, T4, and T6.

Overall, the results indicate that the fiber content in these functional toast samples is influenced by both RF and BSGP levels. Treatments with higher RF content (25%) tend to have higher fiber percentages, and adding BSGP at 1% in combination with RF at 25% (T6) produced the highest fiber content among the samples. These findings suggest that increasing the RF content, particularly in combination with higher BSGP concentrations, has a notable effect on the fiber composition of the toast samples. As reported by previous studies, fortifying bread with dietary fibers from various sources can significantly impact its nutritional profile and quality attributes. They have shown that fiber fortification can increase total dietary fiber content [42] and improve the content of essential minerals in bread [43], aligning with the current study′s findings. However, the source of fiber affects mineral bioaccessibility, with some fibers enhancing and others reducing it [43]. Fiber fortification generally increases dough water absorption and mixing time [44, 45], potentially decreasing loaf volume [44]. The reported impact of fiber on the bread volume will be later validated by investigating the effect of the treatment on the specific volume of the bread. Adding soluble fibers may produce more attractive crumb uniformity and color than insoluble fibers [44]. Fortified bread can exhibit improved textural properties, such as a softer crumb during storage [45]. Additionally, fiber fortification may decrease rapidly digestible starch content and lower the predicted glycemic index of bread [42].

#### 3.1.5. Bread Specific Volume

**Figure 5:**
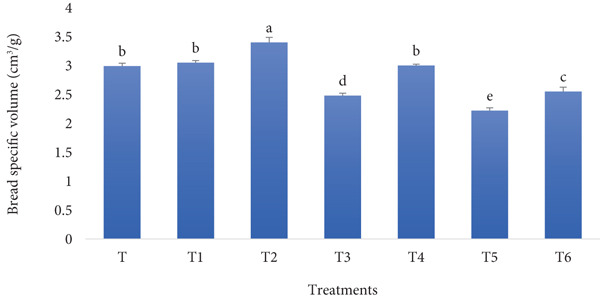
Specific volume of functional bread samples prepared with rye flour and basil seed gum powder. Results are indicated as mean ± SD (*n* = 3). Different characters show a significant difference (*p* ≤ 0.05) among the samples, as assessed by one‐way ANOVA and Tukey′s honestly significant difference tests (T: control, T1: 0% BSGP–15% RF, T2: 0.0% BSGP–25% RF, T3: 0.5% BSGP–15% RF, T4: 0.5% BSGP–25% RF, T5: 1% BSGP–15% RF, T6: 1% BSGP–25% RF).

The specific volume of bread made with fortified flour containing RF and BSGP is generally lower than that of standard wheat bread. The RF contributes less gluten, weakening the dough′s ability to retain gases, while BSGP improves water absorption and dough elasticity but may also create a denser structure if used in high amounts. Figure 5 exhibits the outcome possibly associated with the mentioned justification.

The control sample (T), which contains no RF or BSGP, exhibited a specific volume of approximately 2.8 cm^3^/g. This was statistically different from treatment T2 (0% BSGP–25% RF), which had the highest specific volume at around 3.5 cm^3^/g. Treatments T1 (0% BSGP–15% RF) and T4 (0.5% BSGP–25% RF) showed intermediate specific volumes of about 3.0 and 3.2 cm^3^/g, respectively, with no significant differences from each other but showing slight variation from different treatments. Meanwhile, T3 (0.5% BSGP–15% RF) exhibited a specific volume closer to 2.6 cm^3^/g, statistically lower than the control. Treatment T5 (1% BSGP–15% RF) exhibited the lowest specific volume at approximately 2.2 cm^3^/g, while T6 (1% BSGP–25% RF) had a slightly higher specific volume of around 2.7 cm^3^/g, statistically similar to the control.

Overall, the results indicate that the specific volume of these functional toast samples is influenced by both RF and BSGP levels. Treatments with higher RF content (25%) tend to have higher specific volumes, particularly without BSGP (T2). However, increasing BSGP levels to 1% reduces the specific volume (as seen in T5 and T6). These findings suggest that RF contributes positively to bread volume, but higher concentrations of BSGP may limit the loaf expansion, potentially affecting bread aeration. Previously, iron fortification of wheat bread has been reported to negatively impact quality characteristics, including specific volume, which may decrease by up to 30% [46]. However, fortification with alternative ingredients has also yielded positive results. Adding 5% mealworm powder to bread dough increased specific volume and decreased firmness [47]. Similarly, incorporating *Cordyceps militaris*–fermented chickpea flour at 5% improved specific volume and reduced crumb hardness compared to wheat‐only bread [48]. Finger millet flour fortification at 20% produced bread with acceptable physical properties and enhanced nutritional quality [49]. These studies demonstrate that while some fortification methods may negatively impact bread volume, others can maintain or improve it while enhancing nutritional value. The effects on specific volume depend on the type and amount of fortifying ingredients used, highlighting the importance of careful selection in bread fortification.

#### 3.1.6. Bread Density

**Figure 6:**
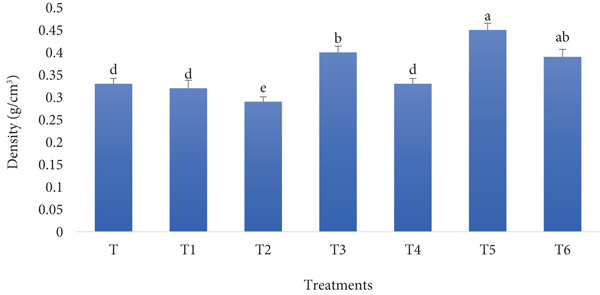
Density of functional bread samples prepared with rye flour and basil seed gum powder. Results are indicated as mean ± SD (*n* = 3). Different characters show a significant difference (*p* ≤ 0.05) among the samples, as assessed by one‐way ANOVA and Tukey′s honestly significant difference tests (T: control, T1: 0% BSGP–15% RF, T2: 0.0% BSGP–25% RF, T3: 0.5% BSGP–15% RF, T4: 0.5% BSGP–25% RF, T5: 1% BSGP–15% RF, T6: 1% BSGP–25% RF).

The density of bread made with RF and BSGP is likely to be higher compared to standard wheat bread due to RF′s lower gluten content, weakening the dough′s gas‐holding capacity, and BSGP, while improving moisture retention and elasticity, can make the dough more compact if overused. As a result, the bread may have a denser structure, with smaller air pockets and a firmer crumb. Figure 6 shows the results of exploring the density of the bread of different treatments, as can be interpreted from the data, fortification of the flour followed by a surge in density.

The control sample (T), which contains no RF or BSGP, exhibited a density of approximately 0.32 g/cm^3^. This was statistically different from treatment T5 (1% BSGP–15% RF), which had the highest density at around 0.41 g/cm^3^. Treatment T6 (1% BSGP–25% RF) followed closely, with a density of about 0.37 g/cm^3^, and was statistically similar to T5. Treatments T1 (0% BSGP–15% RF) and T4 (0.5% BSGP–25% RF) showed intermediate densities of around 0.33 and 0.31 g/cm^3^, respectively, with no significant differences from the control. Meanwhile, T2 (0% BSGP–25% RF) and T3 (0.5% BSGP–15% RF) exhibited the lowest densities, with values around 0.28 and 0.30 g/cm^3^, respectively, significantly lower than the other treatments.

Overall, the results suggest that the density of the functional toast samples increases with the addition of BSGP, particularly at 1% concentration, which is in agreement with a previous study, where it was reported that fortification with debittered and germinated fenugreek seed flour increased the bulk density and crumb firmness and decreased the specific loaf volume [50]. The highest density was observed in treatments containing 1% BSGP and RF, indicating that BSGP significantly influences the bread′s compactness. Conversely, using a higher RF content (25%) without BSGP led to the lowest densities, suggesting that RF contributes to a lighter, less dense bread structure.

#### 3.1.7. Pasting Temperature

**Figure 7:**
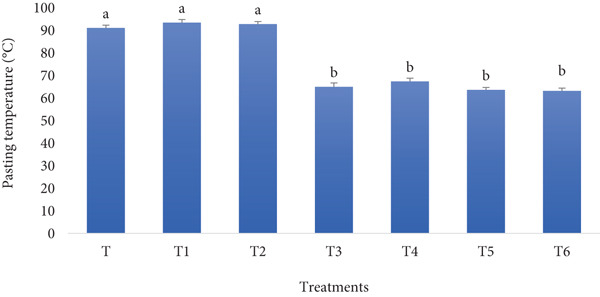
Pasting temperature of functional bread samples prepared with rye flour and basil seed gum powder. Results are indicated as mean ± SD (*n* = 3). Different characters show a significant difference (*p* ≤ 0.05) among the samples, as assessed by one‐way ANOVA and Tukey′s honestly significant difference tests (T: control, T1: 0% BSGP–15% RF, T2: 0.0% BSGP–25% RF, T3: 0.5% BSGP–15% RF, T4: 0.5% BSGP–25% RF, T5: 1% BSGP–15% RF, T6: 1% BSGP–25% RF).

The pasting temperature of bread made with RF and BSGP can increase or decrease depending on their interactions with starch. An increase occurs due to the high‐water absorption of rye pentosans and basil seed gum, delaying starch gelatinization, while a decrease could result from the dilution of starch content and hydrocolloid interactions disrupting starch granule integrity. According to the results of the current study, shown in Figure 7, a significant decrease in pasting temperature can be observed when a combination of RF and BSGP is used for fortification. Noteworthy is the fact that RF solely did not change the pasting temperature compared to the control sample.

The control (T), along with T1 (0% BSGP and 15% RF) and T2 (0% BSGP and 25% RF), exhibited the highest pasting temperatures, all around 90°C–92°C, indicating no significant difference between them. This suggests that the absence of BSGP, regardless of RF content, resulted in similarly high pasting temperatures, likely due to the predominance of wheat starch in the control and the role of rye starch in raising the gelatinization temperature. On the other hand, the samples containing BSGP (T3, T4, T5, and T6) exhibited lower pasting temperatures, ranging from 82°C to 87°C, all of which were statistically different from the control. Introducing 0.5% or 1% BSGP appears to have lowered the pasting temperature significantly across these samples. This can be attributed to the potential interaction of BSGP with water and starch molecules, likely increasing the water availability for starch gelatinization and thus lowering the energy required to reach the pasting point.

Overall, data suggest that including BSGP in bread formulations can significantly reduce the pasting temperature, likely due to its water retention and interaction properties, which facilitate earlier starch gelatinization. The reduction in pasting temperature could have implications for the textural properties of the toast during processing and storage, possibly contributing to changes in moisture retention and crumb structure. RF alone, however, did not significantly impact the pasting temperature in the absence of BSGP. According to previous research, the pasting properties of bread are influenced by various factors, including baking procedures, storage conditions, and fortification. Partial baking, followed by storage and rebaking, affects the pasting temperature, viscosity, and softness of bread crumbs [51, 52]. Adding calcium propionate can decrease pasting temperature while increasing viscosity [51, 52]. Longer initial baking times generally result in lower pasting temperatures and softer crumbs [51, 52]. Extended storage periods decrease the pasting temperature, volume, and softness of rebaked bread [51, 52]. The pH of bread crumbs shows a negative correlation with pasting temperature and peak viscosity [53]. Aligning with our findings, Bankole et al. [54] reported that fortification, such as adding Bambara groundnut flour to gari, can alter pasting properties, with higher fortification levels generally leading to lower peak viscosity and pasting time.

#### 3.1.8. Dough Viscosity

**Figure 8:**
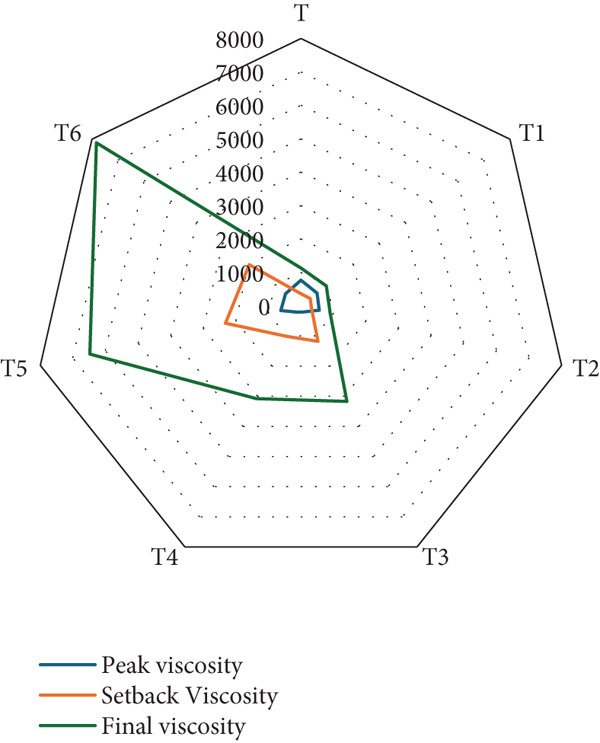
Peak, setback, and final viscosity of functional bread samples prepared with rye flour and basil seed gum powder. The illustrated results are according to mean ± SD (*n* = 3) (T: control, T1: 0% BSGP–15% RF, T2: 0.0% BSGP–25% RF, T3: 0.5% BSGP–15% RF, T4: 0.5% BSGP–25% RF, T5: 1% BSGP–15% RF, T6: 1% BSGP–25% RF).

Figure 8 illustrates the peak viscosity, setback viscosity, and final viscosity of the dough (T1–T6) made with varying proportions of RF and BSGP. These three viscosity parameters are critical in understanding the pasting behavior and structural development of starch‐based systems during heating and cooling. Peak viscosity reflects the extent of starch granule swelling, setback viscosity indicates the degree of retrogradation during cooling, and final viscosity demonstrates the stability and firmness of the final product.

The consistent increase in viscosity values from T1 to T6 suggests that incorporating BSGP plays a key role in enhancing the toast formulations′ water‐holding capacity and structural strength. These findings highlight the role of RF and BSGP in modifying the textural and pasting properties of functional toast. Previous research on fortifying wheat flour with various additives has shown similar effects on dough rheology and bread quality [55].

Among the treatments, T6 exhibits the highest final viscosity, approaching 8000 cP, followed by T5, which shows a substantial increase compared to earlier treatments. This implies that the samples with higher levels of BSGP result in superior gel‐forming ability and increased viscosity stability, likely due to enhanced water retention and structural reinforcement by the hydrocolloid network of BSGP. The moderate peak and setback viscosities suggest that starch swelling is well regulated and gel formation during cooling is robust, which is desirable for maintaining toast texture postbaking. Similar to the current results, previous studies observed that adding sodium stearoyl‐2‐lactylate into soybean and sesame meal–fortified bread significantly increased peak viscosity and improved bread volume, indicating enhanced starch performance under modified formulations [56].

In contrast, Treatments T1 and T2 exhibit the lowest viscosity values across all parameters, suggesting minimal starch gelatinization and poor gel structure. This is likely due to either the absence or very low levels of BSGP, resulting in limited interaction with starch components. Such outcomes align with previous findings where incorporating organic acids into iron‐fortified flour decreased peak viscosity and other viscosity indices, affecting final product quality [55]. Treatments T3 and T4 fall in the midrange of viscosity measurements, indicating that moderate levels of BSGP improve pasting behavior but not to the extent observed in T5 and T6. These patterns are consistent with other studies where fortification with *Auricularia auricula* mushroom flour increased peak and final viscosities but simultaneously altered dough elasticity and stability [57].

Additionally, the progressive increase in viscosity, especially final viscosity, supports the notion that mineral or compound fortification significantly affects dough rheology and pasting performance. In line with this, iron and zinc fortification of whole wheat flour has been shown to alter water absorption, dough development time, and viscoelastic properties, highlighting the delicate balance between composition and functional outcomes [58]. Therefore, the rising trend in final viscosity from T1 to T6 emphasizes that BSGP contributes to water binding and gelatinization and enhances the structural resilience of RF‐based toast. The combination of RF and BSGP proves effective in tailoring pasting behavior, with the higher levels of BSGP in T5 and T6 producing optimal results in terms of viscosity and potential textural performance.

#### 3.1.9. Sensory Attributes

**Figure 9:**
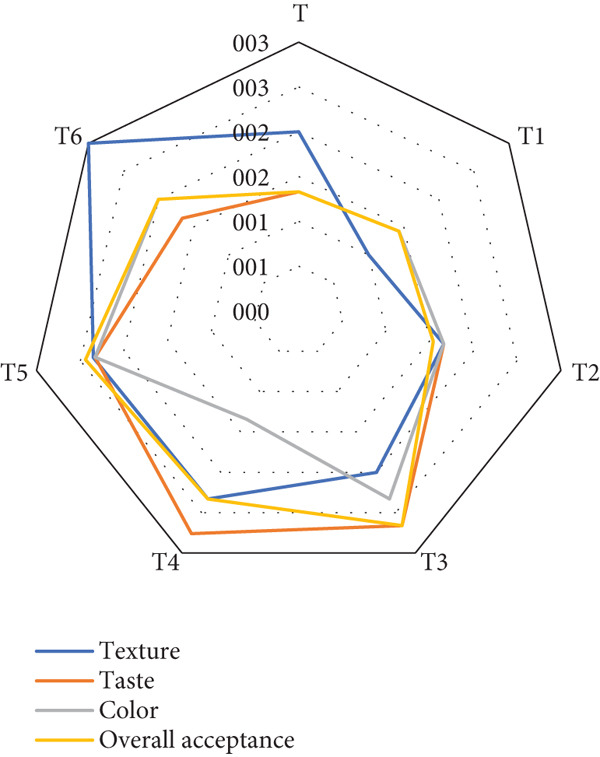
The sensory properties of functional bread samples prepared with rye flour and basil seed gum powder. The illustrated results are according to mean ± SD (*n* = 3) (T: control, T1: 0% BSGP–15% RF, T2: 0.0% BSGP–25% RF, T3: 0.5% BSGP–15% RF, T4: 0.5% BSGP–25% RF, T5: 1% BSGP–15% RF, T6: 1% BSGP–25% RF).

Figure 9 illustrates the sensorial evaluation of functional toast samples formulated with varying levels of RF and BSGP, focusing on texture, taste, color, and overall acceptance. The control sample (T) exhibited moderate scores across all parameters, serving as a baseline for comparison. Among the formulations, T6 (1% BSGP–25% RF) consistently outperformed others, achieving the highest texture and overall acceptance scores, suggesting that this combination positively influences structure and consumer appeal.

In contrast, T4 (0.5% BSGP–25% RF) showed the lowest score in color, indicating a potential visual drawback despite its comparable performance in texture and taste. T1 (0% BSGP–15% RF) and T2 (0% BSGP–25% RF), which lacked BSGP, displayed similar or lower scores across all attributes compared to BSGP‐containing treatments, underscoring the contribution of basil seed gum to sensorial enhancement. T3 and T5, with intermediate BSGP levels (0.5% and 1% BSGP at 15% RF), showed balanced but less pronounced improvements.

Several studies have demonstrated that incorporating nutrient‐dense ingredients can improve the nutritional profile and sensory attributes of bread and toast products. For instance, adding mealworm powder (5%–10%) successfully increased protein content and essential amino acids in bread without compromising technological features, suggesting that fortification can maintain product integrity while enhancing value [47]. Similarly, mixed fermentation of soya flour or rice bran with specific bacteria led to the production of beneficial compounds like vitamin B12 and dextran, which contributed to improved texture, sensory quality, and shelf life, mirroring the improved texture and acceptability observed in treatment T6 [59]. Iron fortification using various compounds did not significantly affect bread′s sensory qualities, though it altered flour characteristics and firmness, indicating that proper formulation can mitigate undesirable changes [60]. Likewise, incorporating finger millet flour (up to 20%) boosted dietary fiber, calcium, and protein while maintaining favorable sensory acceptance [49].

However, the impact of fortification on sensory properties is not uniformly positive across all studies. Iron fortification in wheat bread has been associated with detrimental changes in color, texture, volume, and aroma [47]. However, such effects were milder in gluten‐free formulations, underscoring the importance of matrix interactions. Zinc fortification generally retained consumer acceptance, with minimal reductions in liking scores, particularly in noodles [61]. Additionally, incorporating defatted soy flour at moderate levels (3%–7%) enhanced nutritional value without compromising sensory traits [34], aligning with the balanced improvements in Treatments T3 and T5. The type and level of fortification are crucial, as different iron compounds variably influence bread characteristics [46, 60].

Encouragingly, broader evidence supports that fortified breads can remain sensorially appealing. For example, fortification with up to 20% tilapia fish protein flour retained good taste and improved nutritional quality [36], while products enriched with plant‐based fermented ingredients also received favorable consumer evaluations [31]. *Moringa oleifera* leaf powder showed the best acceptance at 5% fortification, especially in brown bread [62], and zinc‐fortified cereal flours and breads were well received by both children and adults, even at higher levels [63]. These collective findings support the observed enhancements in texture and overall acceptability in toast samples fortified with RF and BSGP, particularly at higher levels (e.g., T6), and justify the viability of such functional formulations in consumer food products.

Overall, the data suggest that BSGP fortification, particularly at 1% in combination with 25% RF (T6), significantly enhanced sensorial qualities, especially texture and overall acceptability, while potentially influencing color and taste. This highlights the synergistic role of hydrocolloids and whole grains in improving functional food properties without compromising consumer acceptance.

### 3.2. Parameters Evaluation Conducted on the Day of Production and the Fifth Day of Storage

**Figure 10:**
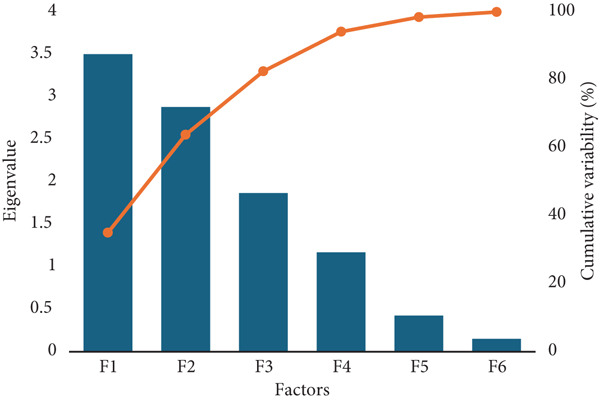
Scree plot illustrating the eigenvalues and cumulative variability of factors (F1–F6) in the analysis. The first two factors (F1 and F2) explain the majority of the variance in the dataset, with diminishing contributions from the remaining factors.

For the evaluation of the quality parameter of the bread on the production day and on Day 5 of postproduction, a PCA was modeled. The scree plot in Figure 10 indicates that the first two factors (F1 and F2) have high eigenvalues, explaining the majority of the variance in the dataset (over 60% cumulatively), while the remaining factors (F3–F6) contribute much less. The orange cumulative variability line levels off after the second factor, suggesting that retaining only the first two would adequately capture most of the data′s variance. This implies that factors beyond F2 provide diminishing returns, making it appropriate to focus on the first two factors for further analysis.

In addition, based on the data provided in Table 2, the contributions of the variables to each factor (F1–F5) vary across different variables (such as moisture, hardness, and adhesiveness). For the first four factors (F1–F4), we observe significant contributions from variables such as Hardness_Day1 and Moisture_Day1, which dominate the first two factors (F1 and F2), explaining a large portion of the variance; for example, Hardness_Day1 contributes 25.848% to F1 and 22.565% to F4, while Moisture_Day1 contributes 15.342% to F1. The variable Brittleness_Day5 also contributes heavily to F5, especially on Day 5, indicating a potentially critical factor for capturing variability in brittleness across the dataset. The selection of factors up to F4 is justified because these factors seem to capture the core variability in the data, including contributions from essential variables such as Hardness_Day1 and Moisture_Day1, without overfitting the model with less significant factors. Thus, F1–F4 encompass a good balance of variability explanation across the key variables.

**Table 2:** Contribution of the variables (%) to each factor (F1–F5) in the analysis. The table shows the percentage contribution of each variable (e.g., moisture, hardness, adhesiveness, springiness, and brittleness) to the respective factors across Day 1 and Day 5.

	**F1**	**F2**	**F3**	**F4**	**F5**
Moisture_Day1	6.190	16.914	4.898	16.743	1.265
Moisture_Day5	15.342	13.099	2.255	0.000	0.000
Hardness_Day1	25.848	1.134	0.159	0.418	1.105
Hardness_Day5	22.565	0.007	0.498	0.412	45.786
Adhesiveness_Day1	0.514	5.649	43.233	0.620	0.752
Adhesiveness_Day5	2.408	25.866	3.216	4.745	4.709
Springiness_Day1	0.830	9.225	35.152	4.028	0.070
Springiness_Day5	0.020	26.250	1.137	15.354	9.457
Brittleness_Day1	5.892	1.205	7.295	50.762	5.001
Brittleness_Day5	20.392	0.651	2.156	6.918	31.855

**Figure 11:**
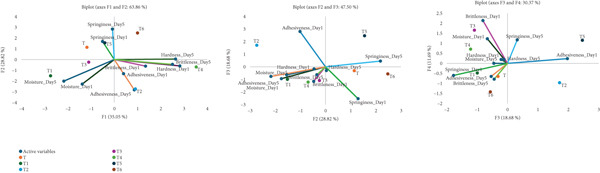
The PCA model of various properties of the bread, explored in two intervals, based on the Pearson correlation matrix, and considering up to five factors. Significant components were selected based on eigenvalues > 1 (Kaiser′s criterion), with variables contributing meaningfully identified by loadings exceeding ±0.5. A significance level of *α* = 0.05 was applied to ensure the statistical validity of the results (T: control, T1: 0% BSGP–15% RF, T2: 0.0% BSGP–25% RF, T3: 0.5% BSGP–15% RF, T4: 0.5% BSGP–25% RF, T5: 1% BSGP–15% RF, T6: 1% BSGP–25% RF).

Figure 11 illustrates the results of a PCA applied to texture attributes of various samples (T, T1–T6) measured on Day 1 and Day 5. These attributes include moisture, hardness, adhesiveness, brittleness, and springiness.

In the first biplot (F1 vs. F2), strong contributions were observed from Moisture_Day1, Moisture_Day5, Springiness_Day5, and Brittleness_Day1, indicating their dominant role in differentiating the samples. Notably, sample T1 aligned closely with Moisture_Day5, suggesting superior moisture retention. This is consistent with findings by Chen et al. [22], where the addition of brewers′ spent grain enhanced water retention and reduced hardness in frozen dough. The cryoprotective behavior of brewers′ spent grain is attributed to its ability to form a more integrated gluten–starch matrix, which reduces free water migration—a possible mechanism also at play in Sample T1. Conversely, Samples T5 and T6, located near vectors representing springiness and hardness, exhibit firmer and more elastic textures. This texture profile can be related to formulations containing ingredients like whey protein concentrate, which has been shown to enhance water absorption and improve dough handling and elasticity when incorporated at optimal levels [64]. The elevated hardness and elasticity observed in T5 and T6 support this, potentially reflecting a protein‐enriched formulation.

The second biplot (F2 vs. F3) emphasizes the roles of Adhesiveness_Day1 and Springiness_Day5. Sample T2, located prominently along this axis, shows high adhesiveness and springiness. This could be attributed to flaxseed flour, which has been reported to increase dough development time and enhance water binding due to its high fiber and protein content [6]. These components contribute to more potent hydration properties, increasing adhesiveness and elasticity. In contrast, Samples T1 and T4, clustering near the negative end of F2 and F3 and aligning with Moisture_Day1 and Adhesiveness_Day5, appear to represent softer, less elastic doughs. This aligns with earlier observations that high moisture retention with limited structural protein reinforcement can yield soft, cohesive textures with lower elastic recovery. Such behavior was also observed in formulations containing dietary fiber‐rich ingredients like *Dictyophora indusiata* powder [4], where moisture increased but specific volume and elasticity decreased.

The third biplot (F3 vs. F4) highlights more subtle distinctions among samples. Here, Brittleness_Day1 and Springiness_Day5 remain influential. Sample T6, located in the negative quadrant, likely exhibits low values for adhesiveness and hardness, which could correspond to a dough matrix disrupted by oversupplementation or an unbalanced ingredient composition. It has been reported that such effects occur at higher levels of fermented whey protein concentrate, where the dough becomes sticky and the structure is compromised [64]. In contrast, Samples T2 and T5 demonstrate strong positive associations with textural strength on Day 5. These samples may represent balanced formulations where water retention and protein structure were optimized, potentially through combinations of ingredients like moderate whey permeate, vegetable pastes, or flaxseed flour. The inclusion of vegetable pastes has been shown to improve nutritional profiles while maintaining acceptable sensory and physical qualities and could explain the favorable elastic properties noted in these samples [5].

The PCA reveals that samples like T1, T5, and T6 exhibit well‐defined trends in texture evolution, notably in moisture retention, firmness, and elasticity. These profiles reflect the potential roles of ingredients with hydration‐modulating, fiber‐rich, and bioactive compound‐containing functionalities. Clustering samples like T3 and T4 near the origin suggests balanced but undistinguished properties, possibly reflecting standard formulations without significant functional enrichment. These findings underscore the value of targeted ingredient selection in optimizing bread product quality.

## 4. Conclusions

This study highlights the potential of incorporating RF and BSGP into functional toast formulations to improve nutritional, rheological, and sensory attributes. Fortification with RF and BSGP significantly enhanced nutritional content: ash increased from 0.85% in the control to 1.35% in T6 (25% RF + 1% BSGP), while fiber rose from 0.9% to 1.4%. Protein content remained relatively stable, with only a slight decrease at higher inclusion levels (T6: 10.1% vs. 10.4% in control). Fat content showed minimal variation but peaked at 7.9% in T6 compared to 7.0% in the control. From a rheological perspective, RF improved specific volume, with T2 (15% RF) reaching 3.5 versus 2.8 cm^3^/g in the control. Conversely, due to its hydrophilic nature, BSGP increased product density (T5: 0.41 vs. 0.32 g/cm^3^) and water retention. Including BSGP also reduced pasting temperature (T6: 82°C vs. 92°C) and increased peak viscosity (T6: 8000 cP), indicating enhanced gel formation and structural stability.

Sensory evaluation identified T6 as the most favorable formulation, excelling in texture, taste, and overall acceptability. Moderate levels of RF and BSGP contributed positively to sensory appeal, while excessive inclusion impaired consumer acceptance. Texture analysis during storage revealed temporal changes: Day 1 samples were moister and springier, whereas Day 5 samples—especially those with higher BSGP—became harder, more brittle, and adhesive. PCA effectively captured these trends, emphasizing the dynamic relationship between moisture, firmness, and elasticity over time.

Overall, the integration of RF and BSGP presents a promising strategy for enhancing the nutritional and functional quality of toast. However, optimal levels must be carefully calibrated to balance fresh product characteristics and shelf life stability. Future research should focus on long‐term storage behavior, including microbial stability and moisture retention, as well as explore the application of RF and BSGP in other baked or gluten‐free products. Understanding consumer preferences across diverse dietary needs will further refine these functional formulations for health‐conscious markets.

## Conflicts of Interest

The authors declare no conflicts of interest.

## Funding

No funding was received for this manuscript.

## Data Availability

The data supporting the findings of this study are available from the corresponding author upon reasonable request.
